# Subacute dislocation of the elbow following Galeazzi fracture-dislocation of the radius: A case report

**DOI:** 10.1186/1752-1947-5-589

**Published:** 2011-12-20

**Authors:** Aysha Rajeev, Shanaka Senevirathna, John Harrison

**Affiliations:** 1 Department of Trauma and Orthopaedics, Queen Elizabeth Hospital, Gateshead, NE9 6SX, UK

## Abstract

**Introduction:**

The Galeazzi fracture-dislocation was originally described by Sir Astley Cooper in 1822 but was named after Italian surgeon Ricardo Galeazzi in 1934. It is an injury classified as a radial shaft fracture with associated dislocation of the distal radioulnar joint and disruption of the forearm axis joint. The associated distal radioulnar joint injury may be purely ligamentous in nature, tearing the triangular fibrocartilaginous complex, or involve bony tissue (that is, ulnar styloid avulsions) or both. We report this case because of the rare association of posterior dislocation of the elbow along with Galeazzi fracture-dislocation. To the best of our knowledge, this has not been previously reported in the English literature.

**Case presentation:**

A 26-year-old Caucasian man presented to our department after a fall from a motorbike. He sustained a closed, isolated Galeazzi fracture-dislocation of the right forearm and no associated elbow injuries, and this necessitated open reduction and internal fixation of the radius. Post-operative radiographs films were satisfactory. However, clinical and radiological evidence of ipsilateral elbow dislocation was noted at a five-week follow-up, subsequently requiring open reduction of the joint and collateral ligament repair. Our patient was noted to have full elbow and forearm function at three months.

**Conclusions:**

Although the Galeazzi fracture-dislocation has been classically described as involving only the distal radioulnar joint, traumatic forces can be transmitted to the elbow via the interosseous membrane of the forearm. This can lead to instability of the elbow joint. Therefore, we recommend that, in every case of forearm fracture, both elbow and wrist joints be assessed clinically as well as radiologically for subluxation or dislocation.

## Introduction

Fractures of the distal third of the radius which are associated with acute distal radioulnar joint (DRUJ) dislocations are known as Galeazzi fracture-dislocation [1]. Originally described by Sir Astley Cooper in 1822, the injury was renamed after Italian surgeon Ricardo Galeazzi, who described it again in 1934. As both the radius and ulna are rigidly constrained proximally and distally, fracture of one bone in isolation is a rarity and usually is associated with dislocations of either the proximal or distal radioulnar joint. Variants of this injury are described in the literature, but the most common is a fracture of the distal third of the radius with DRUJ dislocation, whereas fractures of the proximal third and shaft of the radius are associated with acute elbow dislocations [2-4]. Our report represents an unusual case of fracture of the distal third of the radius with subacute posterior dislocation of the ipsilateral elbow.

## Case presentation

**Figure 1:**
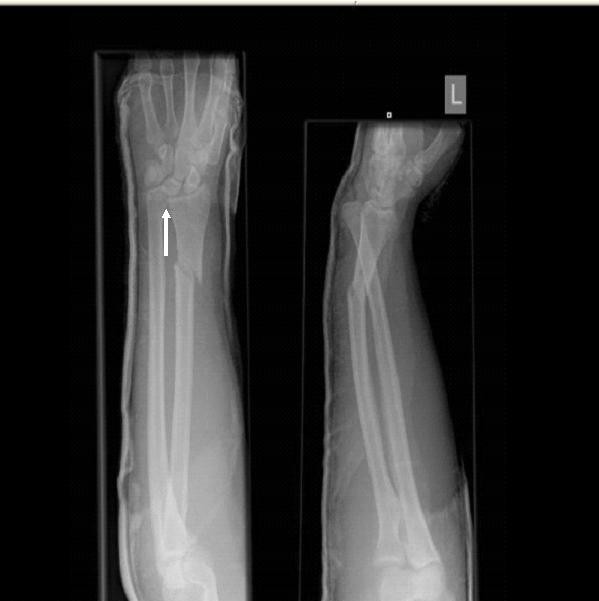
**Radiographs at initial presentation show a comminuted fracture of the right radial shaft (junction of middle and distal thirds) and distal radioulnar joint (DRUJ) dislocation but no evidence of elbow dislocation**.

A 26-year-old Caucasian man presented to our department with an isolated right forearm injury, which he sustained after falling off his motorbike. His main complaint was of a painful and clinically deformed right forearm. A clinical examination revealed a closed and neurovascularly intact injury with bony tenderness and inability to move the right forearm. Radiographs showed a comminuted fracture of the right shaft of the radius (at the junction of the middle and distal thirds) with an associated DRUJ dislocation but no evidence of elbow dislocation (Figure 1).

Our patient underwent open reduction and anatomic fixation of the distal radius fracture with a compression plate via a standard volar Henry approach. His elbow range of movement was checked immediately after fixation, and radiographs confirmed the elbow to be in joint. His elbow had been asymptomatic before the operation and so was not formally tested for instability. After the operation, the limb was rested in a long arm cast with the elbow in 90° of flexion and the forearm in a mid-prone position. The wound was checked at two weeks and was noted to be healing satisfactorily.

**Figure 2:**
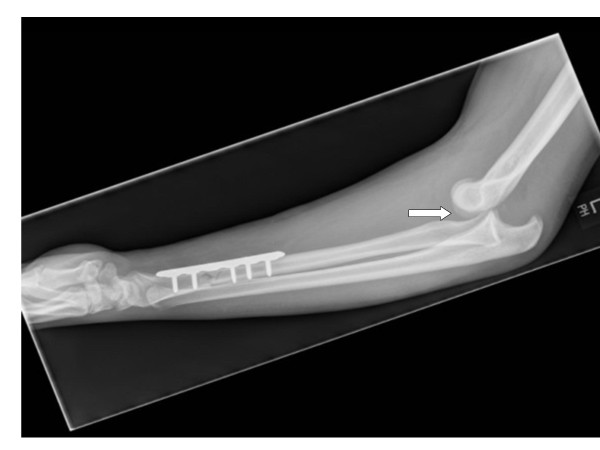
**An image of our patient at five-week follow-up highlights a posteriorly dislocated elbow joint after splint removal**.

**Figure 3:**
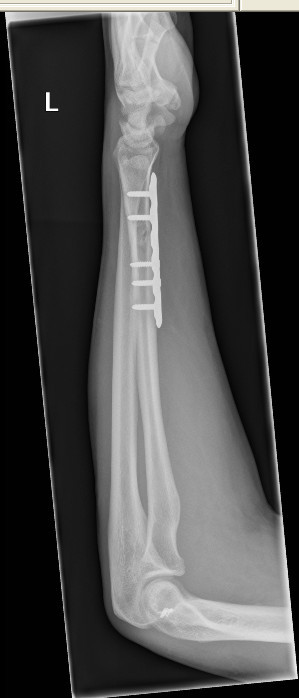
**A postoperative X-ray gives a lateral view after open repair of collateral ligaments**.

**Figure 4:**
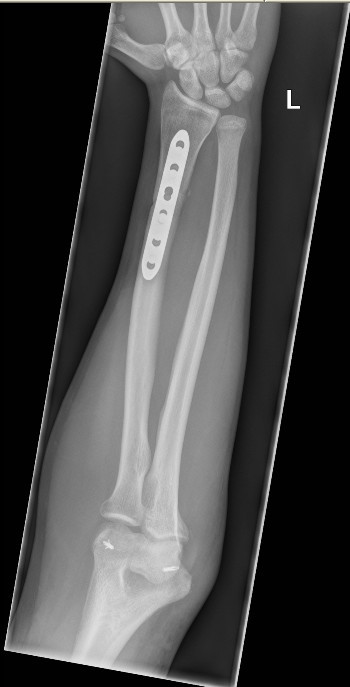
**A postoperative X-ray gives an anteroposterior (AP) view after open repair of collateral ligaments**.

The splint was removed at five-week follow-up, and the elbow joint was clinically and radiologically noted to be posteriorly dislocated (Figure 2). Our patient denied any pain or any other concurrent injuries while being immobilized in the cast. He underwent open reduction and collateral ligament repair (Figures 3 and 4), and the elbow was immobilized in a splint for three weeks after the operation. A full range of movement was achieved at the elbow at three months of follow-up after intensive physiotherapy.

## Discussion

Fractures of the distal third of the radius usually are associated with dislocation or instability of the DRUJ or both and commonly are referred to as Galeazzi fracture-dislocation. The usual mechanism of injury is a fall on an outstretched hand with a pronated forearm at the time of injury. The radius and ulna are parallel bones that are tightly constrained both proximally and distally. As a result, any disturbance in the length of either bone must affect the proximal or distal radioulnar joints or the stability of the elbow joint. The fracture-dislocation injury pattern is dictated largely by the magnitude of the deforming forces imparted and also the position of the upper limb at the time of injury [1].

Although Galeazzi fracture-dislocation historically has been considered a combination of distal radius fracture and DRUJ dislocation, other associated injuries have been described in the literature. Soon and colleagues [4] (1995) described a case of persistent radial head subluxation associated with an ipsilateral radial shaft fracture; the subluxation, missed after the initial injury, was not diagnosed until seven weeks later. Malik and colleagues [5] (2005) described two cases of acute elbow dislocation with radial neck fracture and traumatic DRUJ dislocation, whereas Shiboi and colleagues [3] (2005) reported an unusual case of a posterolateral elbow dislocation with an ipsilateral Galeazzi fracture.

Our case presents a Galeazzi fracture with an associated elbow dislocation, which has yet to be described in the literature. In simple posterior dislocations of the elbow, the mechanism of injury can be thought of as a circle of soft tissue disruption starting from the lateral side and progressing to the medial side in three stages. Stage 1 is characterized by complete disruption of the lateral ulnar collateral ligament complex with partial or complete disruption of the remaining lateral collateral ligament complex. The result is a posterolateral rotatory subluxation (of the elbow) that can spontaneously reduce. Stage 2 includes further disruption resulting in an incomplete posterolateral elbow dislocation with X-rays demonstrating the coronoid process 'perched' on the humeral trochlea. This can be reduced with minimal force. Stage 3 is subdivided into two components. In stage 3A, the posterior part of the medial collateral ligament and all of the soft tissues around it are disrupted whereas the anterior portion, or bundle, is spared. This bundle forms the pivot around which the elbow dislocates posteriorly by way of a posterolateral rotatory mechanism. Stage 3B features complete disruption of all components of the medial collateral ligament complex of the elbow [6].

We speculate that the initial injury in our case not only caused a fracture of the radius but also damaged various capsulo-ligamentous stabilizers of the ipsilateral elbow joint and resulted in posterolateral instability. Although initial radiographs and intraoperative fluoroscopy failed to reveal any elbow instability, we feel that, after fracture fixation and resolution of any soft tissue swelling, the elbow joint dislocated posteriorly.

## Conclusions

The patho-mechanics producing both Galeazzi fracture-dislocation and posterior dislocation of the ipsilateral elbow are strongly correlated. The capsulo-ligamentous structures of both elbow and radioulnar joints are often adversely affected in these types of injuries. We conclude by emphasizing the importance of regular clinical and radiological examinations of both radioulnar and elbow joints in isolated Galeazzi-type fractures.

## Abbreviations

DRUJ: distal radioulnar joint.

## Consent

Written informed consent was obtained from the patient for publication of this case report and any accompanying images. A copy of the written consent is available for review by the Editor-in-Chief of this journal.

## Competing interests

The authors declare that they have no competing interests.

## Authors' contributions

AR prepared the manuscript. SS performed clinical follow-up of the patient and edited the manuscript. JH conducted the surgical management of the patient. All authors read and approved the final manuscript.
